# Germinoma with Involvement of Midline and Off-Midline Intracranial Structures

**DOI:** 10.1155/2014/936937

**Published:** 2014-02-09

**Authors:** Monica Graciela Loto, Karina Danilowicz, Santiago González Abbati, Rafael Torino, Alejandro Misiunas

**Affiliations:** ^1^Division of Endocrinology, Metabolism, Nutrition and Diabetes, 1828 Caseros Avenue, 1152 Buenos Aires, Argentina; ^2^Department of Neurosurgery, British Hospital, 1828 Caseros Avenue, 1152 Buenos Aires, Argentina

## Abstract

Germinomas are malignant intracranial germ tumors, usually found in suprasellar regions. Less than 10% are localized in off-middle structures, and synchronous involvement of both structures has only exceptionally been published. A case of an 18-year-old male patient with progressive right-sided hemiparesis and panhypopituitarism was reviewed. Brain MRI showed a solid mass involving pituitary and hypothalamus with thickening of pituitary stalk, high intensity lesions on T2-weighted imaging in left internal capsule, caudate nucleus, globus pallidus, and mild atrophy of the left internal capsule and cerebral peduncle. Nonadenomatous lesions were considered in the differential diagnosis. Alfa-fetoprotein (AFP) levels were negative in both serum and cerebrospinal fluid (CSF), while **β**-human chorionic gonadotrophin (**β**-HCG) levels were slightly increased in CSF. A transsphenoidal biopsy identified a germinoma. Four cycles of chemotherapy with bleomicine, etoposide, and cysplatin were given, followed by radiotherapy, but patients died due to a recidiva. *Conclusion*. Germinoma must be considered in patients with insipidus diabetes with a sellar mass with thickening of pituitary stalk; and ectopic germinoma must be suspected in patients with slowly progressive hemiparesis with cerebral hemiatrophy. Even with a rare condition, colocalization of midline and off-midline germinoma must be suspected in the presence of these typical signs of both localizations.

## 1. Introduction 

Germ cell tumors (GCT) represent approximately 3% of neoplasms in children's cancer registries [1]. They constitute 0.1 to 2.4% of all childhood intracranial tumors in North America and Europe, while they account for almost 2.1 to 9.5% in Japan and the Far East [2, 3]. Central nervous system germ cell tumors (CNSGCT) are rare and most of them occur in patients under 20 years of age [1, 4].

CNSGCTs have been classified in “secreting” and “nonsecreting” tumors. Secreting tumors are defined as those presenting with an elevated CSF AFP ≥ 10 ng/mL or above the local laboratory's normal range and/or a CSF *β*-HCG level ≥ 50 IU/l or greater than the accepted laboratory normal range. This has been shown to be related to prognosis and treatment response [1]. Brain germinomas are usually serologically negative for these markers [5].

The most common sites of involvement of intracranial germinomas are the pineal or suprasellar regions, while some patients have both localizations at the time of diagnosis [1, 4]. Off-midline germinomas arising in the basal ganglia, thalami, and internal capsule, also called ectopic germinomas, are rare entities representing only 5 to 10% of all CNSGNC [4, 6].

We describe an unusual case of a male patient with a germinoma with a synchronous involvement of midline and off-midline structures. Such a case has only been described twice to our knowledge [4, 7].

## 2. Case Report

An 18-year-old male patient was admitted to the hospital with psychomotor excitement, polyuria, polydipsia, vomits, and a seven-month history of progressive right-sided hemiparesis with dystonia. He had poor school performance, anxiety, and emotional lability for the last two years. Brain nonenhanced computed tomography done seven months before admission was normal. On physical examination, he was in a poor general condition, pale, with a low low body mass index (14,4 kg/m^2^). Blood pressure was 90/60 mmHcg, with a poor response to fluid administration. He had a Tanner-stage 3, with 6 mL testis, pubic hair: G-2, and axillary hair: G-2. On neurological examination he presented a right hemiparesis with hyperreflexia and dystonia.

The presence of polyuria with low urine density in association with hypernatremia suggested the diagnosis of diabetes insipidus (DI). The refractory arterial hypotension suggested adrenal insufficiency. Intravenous hydrocortisone was then started. Afterwards, levothyroxine and oral desmopressin acetate were added. Laboratory examination confirmed the diagnosis of hypopituitarism (Table 1). Clinical response was evident, with a dramatic improvement after hormonal substitution. However, normal natremia levels were difficult to achieve, and desmopressin dose was adjusted. Right-sided hemiparesis persisted, and a program of physical rehabilitation was promptly started. Vomits also persisted, although less frequently.

**Figure 1:**
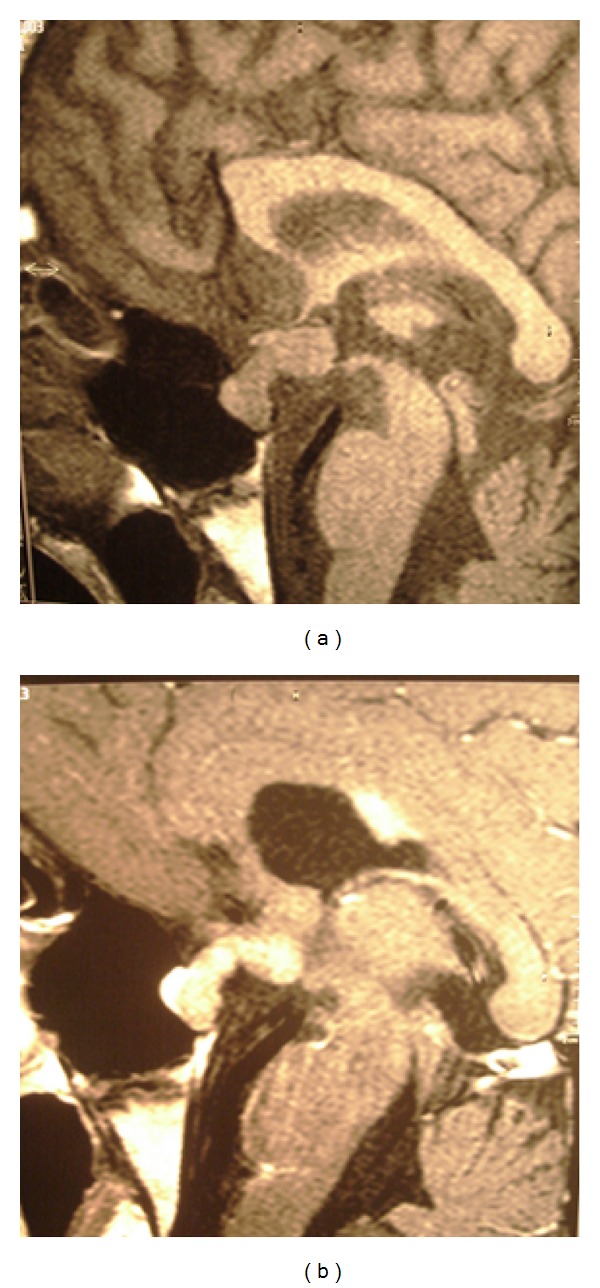
Solid mass involving hypophysis and hypothalamus with marked thickening of the pituitary stalk (a), with enhancement after gadolinium (b).

**Figure 2:**
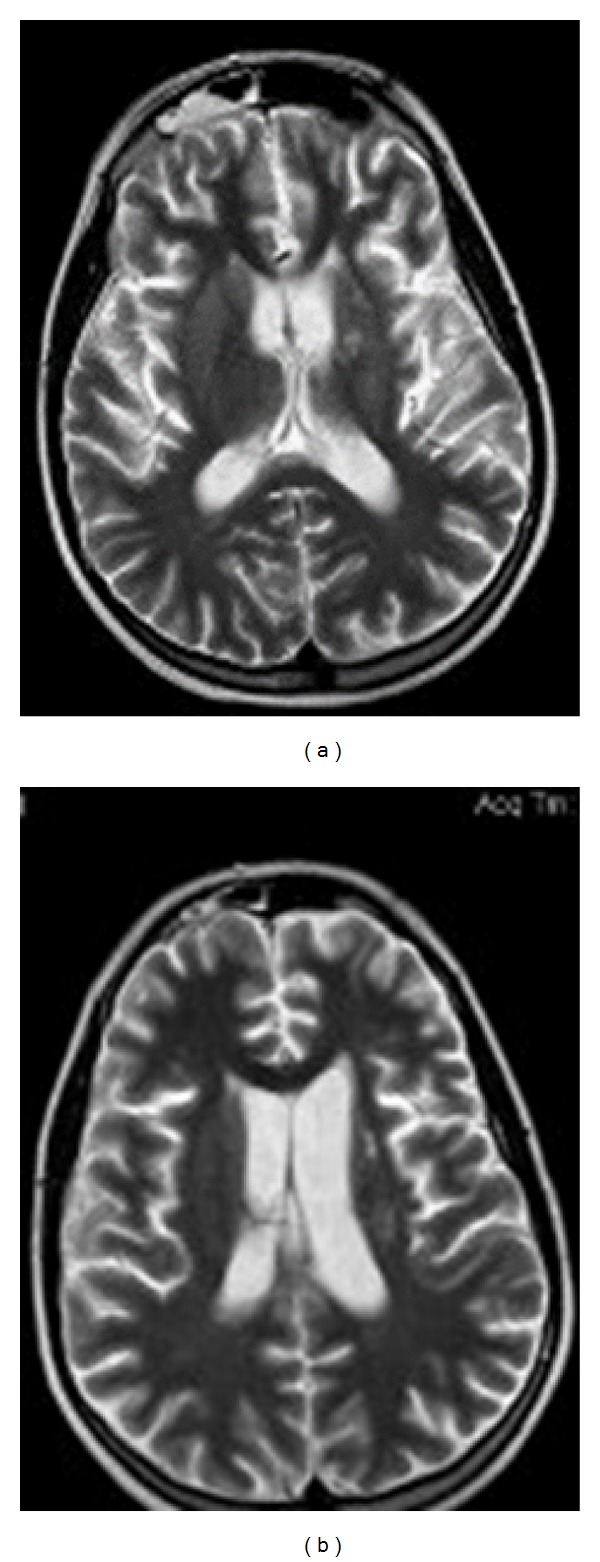
T2-weighted images reveal hyperintense lesions over the left internal capsule (a) and over corona radiata (b).

**Figure 3:**
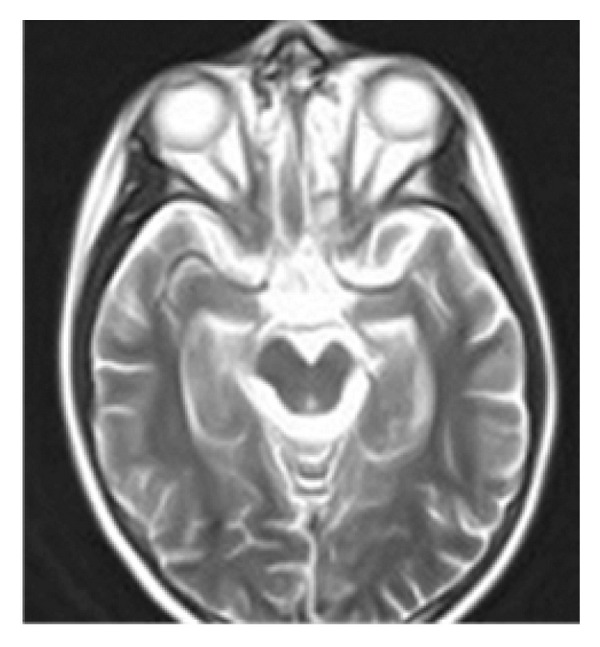
T2-weighted images reveal an atrophic left midbrain peduncle.

Brain magnetic resonance imaging (MRI) showed a solid mass with homogeneous enhancement after gadolinium, involving the pituitary and the hypothalamus with marked thickening of the pituitary stalk. The posterior pituitary hyperintensity was absent on T1-weighted images (Figure 1). A high signal intensity lesion on T2-weighted images was evident in the left internal capsule, corona radiata, caudate nucleus, and globus pallidus bilaterally (Figure 2). Mild atrophy of the left internal capsule and homolateral cerebral peduncle was also evident (Figure 3).

With the suspicion of germinoma, a lumbar punction was indicated. Differential diagnosis with other nonadenomatous lesions was also considered: normal thoracic TC and angiotensin converting enzyme ruled out sarcoidosis; skull and long bones X-rays were normal, without typical lytic lesions of Langerhans cell histiocytosis. The CSF examination did not show atypical cells. AFP levels were negative in both serum and CSF, while HCG was slightly increased only in CSF. A transsphenoidal biopsy identified a pure germinoma. A spine MRI excluded metastatic lesions.

A treatment of four cycles of chemotherapy every three weeks with bleomicine, etoposide, and cysplatin was given.

**Figure 4:**
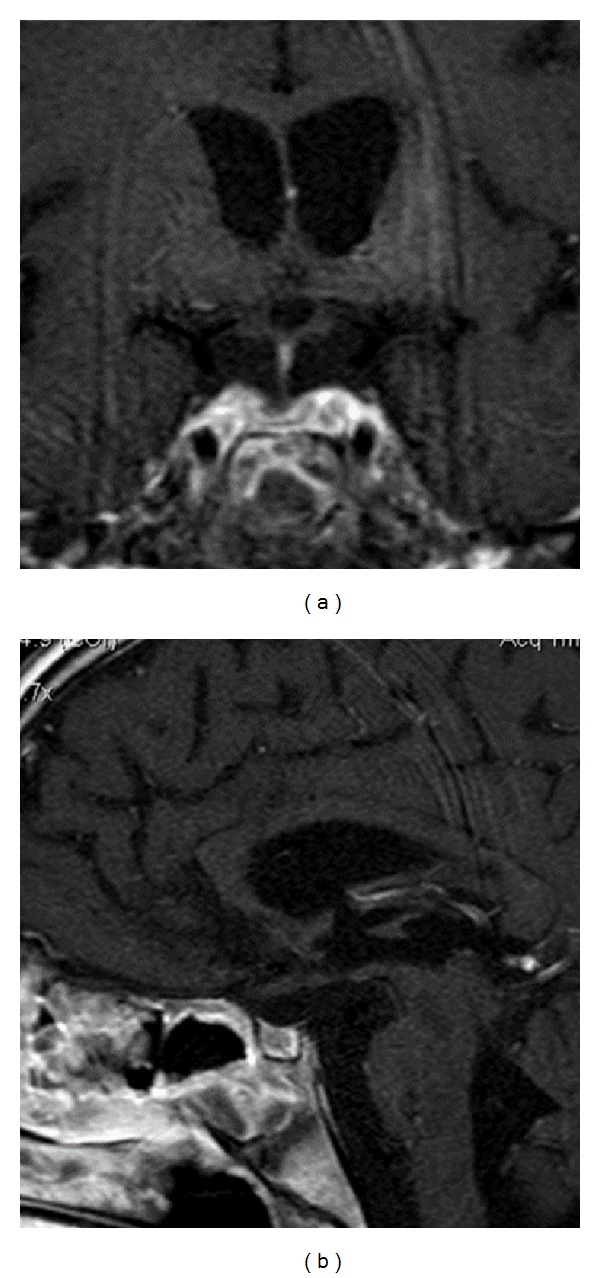
Axial T1-weighted (a) and sagittal T1-weighted (b) MRI after gadolinium enhancement one month after chemotherapy, MRI showed resolution of pituitary-hypothalamic mass, with a marked reduction in stalk-thickness.

An MRI study a month after chemotherapy showed a complete response with disappearance of the pituitary and suprasellar mass (Figure 4), while a reduction in off-midline white matter lesions was also evident. Three months later MRI had no changes, showing no evidence of tumoral recurrence.

After an improvement in his general condition, whole brain radiation therapy was indicated nine months after chemotherapy. Unfortunately, he died after a thalamic recurrence shortly after concluding this last treatment.

## 3. Discussion

Germinomas are the most common and least malignant intracranial germ tumors, usually found in the pineal and suprasellar regions. Five to 10% of the GCTs are ectopic, being localized in off-middle structures, like the thalamus, basal ganglia and internal capsule. Synchronous involvement of midline and off-midline structures, as described in this case, has only exceptionally been published [4, 7]. Clinical presentation depends upon the size and the localization of the tumor. Patients with suprasellar GNC usually present hypothalamic-pituitary dysfunction, being DI one of the most common symptoms, isolated or in association with other hormone deficiencies. Ophthalmic abnormalities such as bilateral hemianopsia may also be present [4]. In our case hypopituitarism with DI was found. Suprasellar germinoma must be suspected in all young patients with isolated DI or in association with other pituitary deficits even when neurological and ophthalmological symptoms are absent [5]. Nevertheless, differential diagnosis with nonadenomatous inflammatory (infundibuloneurohypophysitis, sarcoidosis, and Wegener granulomatosis), neoplastic (Langerhans cell histyocitosis, craniopharyngiomas, metastases, leukemias, lymphomas, and brain tumors), or infectious (tuberculosis) lesions involving pituitary stalk must be considered [6, 7]. It is important to emphasize that the presence of DI almost rules out a pituitary adenoma.

Clinical manifestations of ectopic germinomas are insidious. Slowly progressive hemiparesis and neuropsychiatric symptoms such as dementia, psychosis, or cognitive decline with poor school performance are usually found; cognitive abnormalities are usually one of the earliest manifestations [4, 8–14]. In one of the most recently published series including 20 patients with basal ganglia or thalamic germinomas, all of them had hemiparesis at the time of diagnosis and 45% had cognitive decline [9]. Duration of clinical symptoms ranged from 1 month to 4.5 years, with a mean period of 1.5 years [11]. In accordance with the aforementioned, in our patient a progressive cognitive impairment and a seven-month history of progressive neurological deficit were evident.

Neuroimaging studies are useful in differential diagnoses. But measurement of serum and CSF tumor markers and/or histological studies are required for the confirmation of the diagnoses of germinoma. In this case the MRI showed a solid sellar and suprasellar mass with marked thickening of the pituitary stalk tumor (in accordance with panhypopituitarism), in addition to high signal intensity lesions in the left internal capsule, caudade nucleus, and both globus pallidus with atrophy of the left internal capsule and cerebral peduncle, confirming the synchronous involvement of midline and off-midline structures (in accordance with hemiparesis and cognitive decline). In the early course of the disease, before the appearance of motor symptoms, MRI changes are not so evident. The earliest and most common feature on MRI is the atrophy of the basal ganglia as well as the presence of subtle signal intensity changes as hyperintensity on T1- and T2-weighted images [13]. In our patient, the synchronous presence of the sellar mass was a clue for the presumptive diagnosis of ectopic germinoma.

A tumor biopsy is required for the diagnosis, except in cases where tumor markers are elevated [1]. In our case, both tumor markers were negative in serum and *β*-HCG was slightly increased in CSF. A transsphenoidal biopsy settled the diagnosis of a pure germinoma being negative for both *β*-HCG and AFP, but positive for placental alkaline phosphatase (PLAP). However, biopsy-proven germinomas can have nongerminomatous elements among the unbiopsied sites and nonsecreting tumors can also have nongerminomatous components with a less favorable prognosis [14]. This was probably the case in our patient, considering his bad evolution in a short period of time.

Early diagnosis is very important because a delay in treatment can result in more severe neurologic deficits, as observed in our case. It has been demonstrated that, except for patients with small tumors, pituitary dysfunction before treatment persists or even worsens after tumor remission, mainly after radiotherapy. The earlier diagnosis and the prompt starting of treatment, before irreversible pituitary-hypothalamic damage occurs, contribute to improving the outcome of pituitary function in patients with neurohypophyseal germinomas [10]. In our case, even when only chemotherapy was given initially, panhypopituitarism and hemiparesis persisted after treatment.

The optimal management strategy for CNSGCTs remains unsettled due to a lack of prospective trials, mainly due to the infrequency of these tumors [14]. Germinomas are extremely sensitive to both irradiation and platinum-based chemotherapy but the recurrence rate after initial therapy may be approximately 10% or higher [1, 14–17]. Standard treatment for germinomas has been craniospinal irradiation (CSI) with survival rates of more than 90%. In order to avoid relapses, high dose radiotherapy delivered to the whole ventricle or a larger field is necessary. In an attempt to reduce the morbidity of CSI, cooperative groups had investigated the feasibility of a sequential treatment of chemotherapy followed by focal irradiation [15, 16] or even chemotherapy alone. This last approach was tested by the International Central Nervous System Germ Cell Tumor Study Group, reporting a high rate of complete response but with a high rate of relapse [17]. However, response to irradiation after recurrence is usually very good [18, 19]. Chemotherapy combined with reduced-dose radiation therapy has shown promising results in the tumor control [14–17, 20–24]. But longer follow-up periods are necessary to draw firm conclusions regarding the superiority of this treatment over standard-dose [25], considering that a late recurrence is not a rare event [26].

In our patient, a treatment of four cycles of chemotherapy with bleomicine, etoposide, and cysplatin was chosen, with a very satisfactory initial response. Coadjuvant radiation therapy was administered nine months after, but he died because of a thalamic recurrence of the tumor.

In conclusion, sellar germinomas must be ruled out in all young patients with isolated DI or in association with other pituitary deficits. Ectopic germinoma must be suspected in patients with insidious neuropsychiatric symptoms and progressive hemiparesis, particularly if it is associated with subtle focal lesions in the basal ganglia and cerebral hemiatrophy. Though infrequent, involvement of both midline and off-midline structures may be present. A prompt diagnosis can avoid clinical sequelae and diminishes long-term impairment. The optimal therapeutic strategy for CNS-GCTs is not established yet, and an individualized approach is recommended.

## Figures and Tables

**Table 1:** Laboratory evaluation on admission (normal values in parentheses).

Laboratory parameters	
TSH	2.2 mUI/L (0.5–5 mU/mL)
Triiodothyronine (T3)	76 ng/dL (70–190 ng/dL)
Thyroxine (T4)	5.1 ug/dL (5–12 ug/dL)
Free thyroxine	8 pmol/L (9–26 pmol/L)
Antithyroperoxidase antibodies	Negative
PRL	88.5 ng/mL (2–15 ng/mL)
IGF1	34 ng/mL (163–584)
GH	0.1 ng/mL
LH	<0.2 UI/L (1.3–13 UI/L)
FSH	1.2 UI/L (0.9–15 UI/L)
Testosterone	0.1 ng/mL (3–10 ng/mL)
Cortisol	2.5 ug/dL (5–21 ug/dL)
